# Phenotyping of isolated mesh associated pain secondary to continence mesh device insertion

**DOI:** 10.3389/fpain.2026.1793212

**Published:** 2026-06-17

**Authors:** Hawra Badri, Karen Ward, Richard Edmondson, Fiona Reid

**Affiliations:** 1 The University of Manchester, Manchester, United Kingdom; 2 Health Innovation Manchester, Manchester, United Kingdom; 3 Manchester University NHS Foundation Trust, Manchester, United Kingdom

**Keywords:** mesh, neuropathic, nociceptive, nociplastic, pain, phenotype

## Abstract

**Introduction:**

Isolated Mesh associated Pain Syndrome (I-MAPS) is the most common complication of continence mesh devices. The pain mechanism involved remains unknown. The aim of this study is to characterise the phenotype of women with I-MAPS related to a single continence device and to determine the pain mechanisms involved. These findings are intended to support better understanding of the management of I-MAPS.

**Study design and methods:**

A cross-sectional study set within a quaternary-level mesh complications service. All women with I-MAPS related to a single continence device were included. Pain phenotype was determined using pain body-mapping, the Pain DETECT questionnaire (PDQ), and the Electronic Patient Assessment Questionnaire (e PAQ) to measure vagina and bladder pain. Quality of Life (QOL) and mental wellbeing were ascertained using the EuroQol group-5 Dimension (EQ5D) and WHO-5 wellbeing index.

**Results:**

Over 5-years, 280 women presented with I-MAPS. Subjects reported high levels of pre-existing pain-inducing conditions (*n* = 146/280 52%). Body-mapping of pain identified localised pain suggestive of nociceptive pain and neuro-anatomically distributed pain consistent with neuropathic pain. Two percent (*n* = 5/280) of patients reported distant pain suggestive of a nociplastic component. PDQ identified the largest patient group had neuropathic mediated pain (*n* = 78/142 55%). Twenty-two percent (*n* = 31/142) had nociceptive pain and the remaining 23% (*n* = 33/142) exhibited pain of mixed origin. Trans-obturator (TOT) devices were associated with a significantly higher mean PDQ score of 20 compared to a mean of 17 in retropubic devices (*p* = 0.04). There was a reported, “moderate” impact in ability to perform usual activities of daily living and “moderate” rates of anxiety and depression across the study group. Poor physical and mental well-being scores were reported in patients with I-MAPS of 53/100 and 28/100 respectively.

**Conclusion:**

I-MAPS appears to be of neuropathic phenotype with evidence of mixed origin. Nociplastic features were identified including functional disability and reduced mood and QOL. These exemplify the multidimensional impact and burden of chronic pain.

## Introduction

Surgery using synthetic devices made from polypropylene mesh were once the most common Stress Urinary Incontinence (SUI) procedures performed in the United Kingdom (U.K) spanning over two decades. These included retropubic (Tension Free Vaginal Tape) or Trans Obturator (TOT) sited pelvic mesh devices. Since 2018, surgical management of Stress Urinary Incontinence (SUI) using mesh has been subject to restriction in some countries due to growing concerns regarding their safety profile (1). Mesh infection, perforation into surrounding organs and vaginal exposure were amongst the complications reported. However, chronic pain associated with mesh devices has emerged as the most prevalent complication and associated with debilitating consequences for affected women (2–4). This pain can occur in conjunction with bladder, bowel, vaginal and sexual pain. Recognising the constellation of symptoms and uncertain pathophysiological mechanism associated with this pain, the European Association of Urology introduced the term Mesh associated pain syndrome (MAPS) (5) to describe this condition. We have termed MAPS without other co-existing mesh complications, Isolated Mesh Associated Pain Syndrome (I-MAPS).

Whilst much of the research efforts related to I-MAPS have focused on management, there has been little focus on identifying the cause or defining the mechanism involved in I-MAPS (6). The pathophysiology of pain following continence mesh device insertion remains unknown.

Pain phenotyping is a method of classifying pain based on patient experience, observable traits, psychological factors and prognostic indicators (7). Pain phenotyping allows patients with these characteristics to be divided into pain subgroups, for their pain type to be more precisely defined and more effective, phenotype-specific treatment to be offered.

Pain phenotyping is particularly valuable in conditions of uncertain aetiology and can provide an alternative classification of pain to “non-specific pain” which can be stigmatizing (8) and can generate a nocebo response to treatment.

The International Association for the study of pain (IASP) (9) describes the mechanism of pain as arising from 3 sources. These include nociceptive, arising from actual or threatened tissue injury producing localised pain. Neuropathic related to disease or injury of the peripheral nervous system presenting within a neuroanatomically plausible distribution. More recently the IASP have introduced a third pain term of nociplastic pain. Nociplastic pain is mechanistically defined as pain occurring secondary to altered sensory pathways within both the peripheral and central nervous system (CNS) resulting in heightened pain sensitivity and pain presenting beyond a neuroanatomically related distribution. Nociplastic pain has been used to define chronic pain conditions which do not exhibit obvious nociceptive or neuropathic characteristics.

The aim of this study is to explore the pain phenotypes involved in I-MAPS in patients with continence devices, to examine if differences exist in the pain mechanisms of differing continence devices and to attempt to define the pain phenotype of I-MAPS to support better understanding of its management.

## Methods

All patients with I-MAPS related to a single SUI mesh device (retropubic and trans obturator) presenting to our quaternary-level mesh complications service between 26/01/2018 and 19/04/2024 were included in the study. Patients with Pelvic Organ Prolapse (POP) mesh devices, multiple mesh devices or any other mesh complication were excluded.

Data were collected from the electronic patient records. All data and assessments were collected and performed as part of routine clinical care hence Research Ethics Committee (REC) approval was not required.

Data collected included demographics and details of the mesh device.

### Pain assessments

Body maps were utilised to illustrate the extent and anatomical locations of pain as described by patients or indicated by them on a body outline. Patients were asked to complete the Pain DETECT questionnaire (PDQ) (10). The PDQ is a validated 9-point, patient-rated pain assessment for the screening of neuropathic pain components. It generates a score of between 0 and 38. A score below 12 suggests nociceptive pain phenotype with unlikely neuropathic pain component (<15%). Scores between 13 and 18 (equivocal) suggest neuropathic-mediated pain with possible nociceptive components (mixed aetiology). A score greater than or equal to 19 carries a high probability (>90%) of having neuropathic origins.

All patients were invited to complete the Electronic Patient Assessment Questionnaire (e PAQ). This a validated web-based assessment tool providing self-reported symptoms and quality of life (QOL) data including pain affecting bladder, bowel, vagina and sexual function. Symptoms are scored from 0 to 100, with increasing intensity of pain generating a higher score.

### Assessment of quality of life (QOL)

QOL was assessed using the EuroQol group-5 Dimension (EQ5D) and the World Health Organization-five Wellbeing index (WHO-5 wellbeing index). These questionnaires allow patients to self-report symptoms including functional abilities, mental wellbeing and overall health status.

The EuroQol group-5 Dimension (EQ5D) questionnaire (11), is a standardised measure of health-related quality of life including ability to mobilise, undertake self-care, usual activities, and self-assessment of mental and physical well-being. A score of 1 denotes having no problems with these activities. A score of 2 a slight problem. A score of 3 a severe problem. A score of 4, being unable to perform the activity at all.

Physical wellbeing was subjectively scored out of 100 using a Visual Analogue Score (VAS) (12). A score of 0 describes “the worst health imaginable”, and a score of 100 “the best health imaginable”.

The WHO-5 wellbeing index (13) is a subjective screening tool to identify potential depressive symptoms. It measures mood over a 2-week period using five positively worded statements rated on a 6-point scale. After multiplying the raw score by 4, a final score from 0 (worst imaginable well-being) to 100 (best imaginable well-being) is obtained. A score of 50 or less indicates poor well-being, and a score of 28 or below suggests possible depression.

The e PAQ questionnaire (14) was used to assess the impact of bladder, bowel, vaginal and sexual symptoms on QOL. Impact on QOL is scored from 0 to 100, with increasing impact on QOL generating a higher score.

Demographic data, pain assessments and QOL were examined and compared between patients with retropubic and trans obturator devices and by pain sub-group.

### Statistical analysis

Analysis was performed using Prism (version 10.5.0, Graphpad, San Diego, CA, USA). D’Agostino Pearson test was used to assess data for normality. Fishers exact test was performed to assess categorical data. Un paired *t* test was used to compare normally distributed data. Non-normally distributed data was compared using Mann–Whitney *U* test or Wilcoxon ranked test when relevant. One-way Annova was used to determine differences between the 3 study groups in normally distributed data and multiple comparisons were undertaken using Tukey's test. Kruskal–Wallis test was performed for 3-way analysis of non-parametric data. *P* values <0.05 were considered to indicate statistical significance. We acknowledge the potential inflation of Type I error due to multiple testing; however, unadjusted *p*-values <0.05 are reported to highlight potentially meaningful findings.

## Results

The demographic data are presented in Table 1. Over 5 years, 280 women were managed with I-MAPS related to a single continence device. Women were predominately post- menopausal (median age 60) and obese (median BMI 30). The cohort had a high rate of co-existing chronic pain-inducing conditions (52% *n* = 148/280). Women with trans obturator devices had significantly higher chronic pain conditions, than those with retropubic devices. The median Index of Multiple Deprivation (IMD) based on post code for patients with I-MAPS was 5, indicating that deprivation within this cohort was within the middle of the spectrum. IMD ranks neighbourhoods in England from 1 (most deprived area) to 10 (least deprived area) based on indices including educational attainment and income (15).

**Table 1:** Demographic data.

Patient demographics	Total (*n* = 280)	Retropubic (*n* = 145)	Trans obturator (*n* = 135)	*P* value
Age Median (Range) [IQR]	60 (33–94) [57–60]	60 (38–86) [58–61]	60 (33–46) [56–61]	0.72^b^
BMI Median (Range) [IQR]	30 (19–47) [26–34]	30 (19–47) [26–34]	30 (22–47) [26–35]	0.76^b^
Index of deprivation Median (Range) [IQR]	5 (1–10) [3–8]	5 (1–10) [2–8]	6 (1–10) [3–10]	0.14^b^
Smoking (%)	26 (9%)	17 (12%)	9 (7%)	0.16^a^
Co-existing Chronic Pain conditions (%)	146 (52%)	67 (46%)	79 (59%)	**0** **.** **04** ^a^
Autoimmune conditions (%)	25 (9%)	11 (8%)	14 (10%)	0.53^a^
Mood conditions (%)	45 (16%)	21 (14%)	24 (18%)	>0.99^a^
Diabetes (%)	23 (8%)	11 (8%)	12 (9%)	0.83^a^

a Fishers exact test.

b Mann–Whitney *U* test.

Bold value is statistically significant.

### Pain assessments

#### Body mapping of reported pain

**Figure 1:**
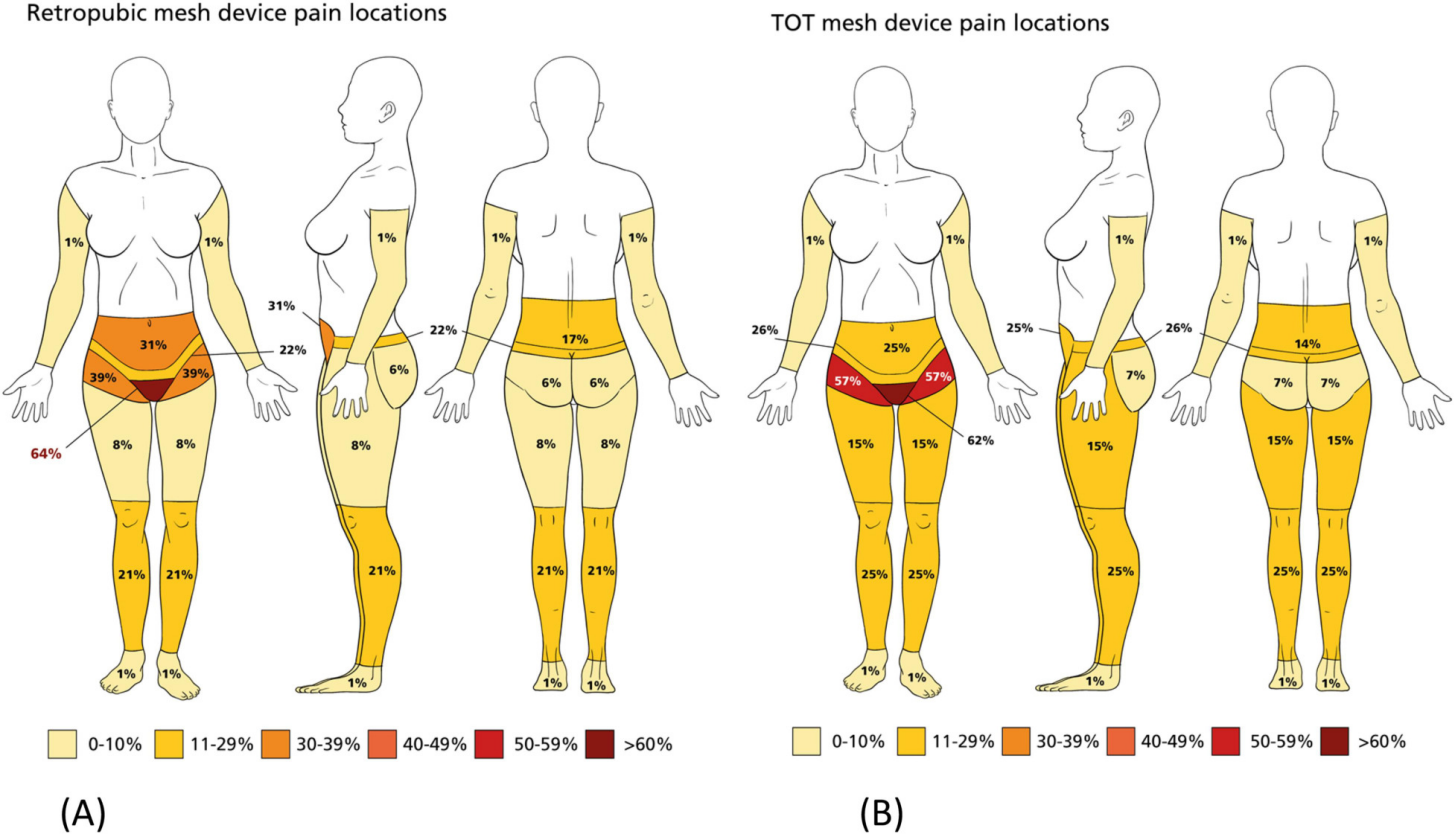
**(A)** Pain locations of patients with retropubic mesh devices (*n* = 145). **(B)** Pain locations of patients with trans obturator mesh devices (*n* = 135).

The anatomical locations of pain by mesh device in the 280 patients seen with I-MAPS is illustrated in Figure 1. Localised pain (vagina, groin and abdomen) was reported in 25%–64% of patients with both retropubic and trans obturator devices. Radiation of pain from the site of device insertion to regions with shared nerve pathways (thighs and legs) were experienced by 8%–25% of patients. Pain beyond the expected neuroanatomical distributions (feet and arms) were reported in 2% of patients (*n* = 5/280). Patients with TOT devices reported significantly higher rates of groin pain than those with retropubic tapes (57% v 39% *p* = 0.03) (Figure 3B).

**Figure 2:**
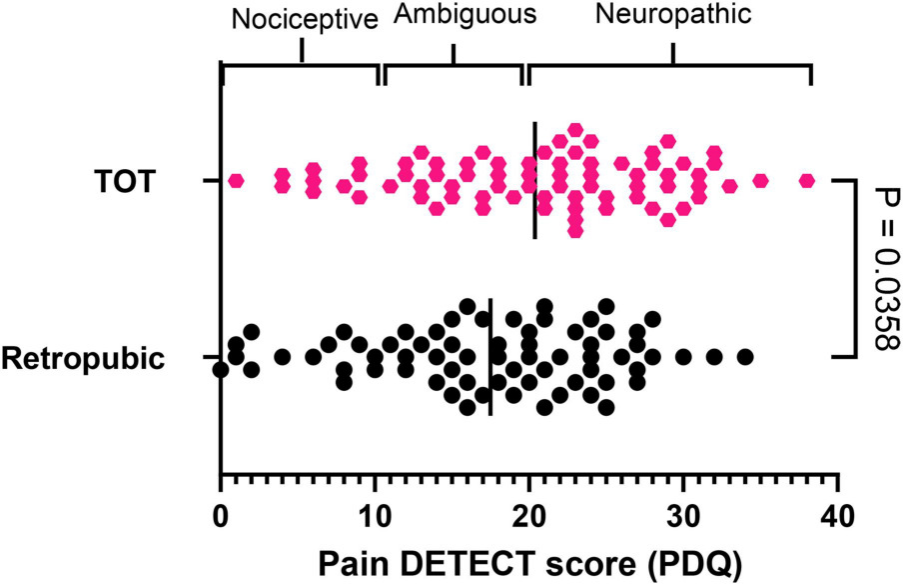
Comparison of PDQ scores between retropubic and trans obturator devices.

**Figure 3:**
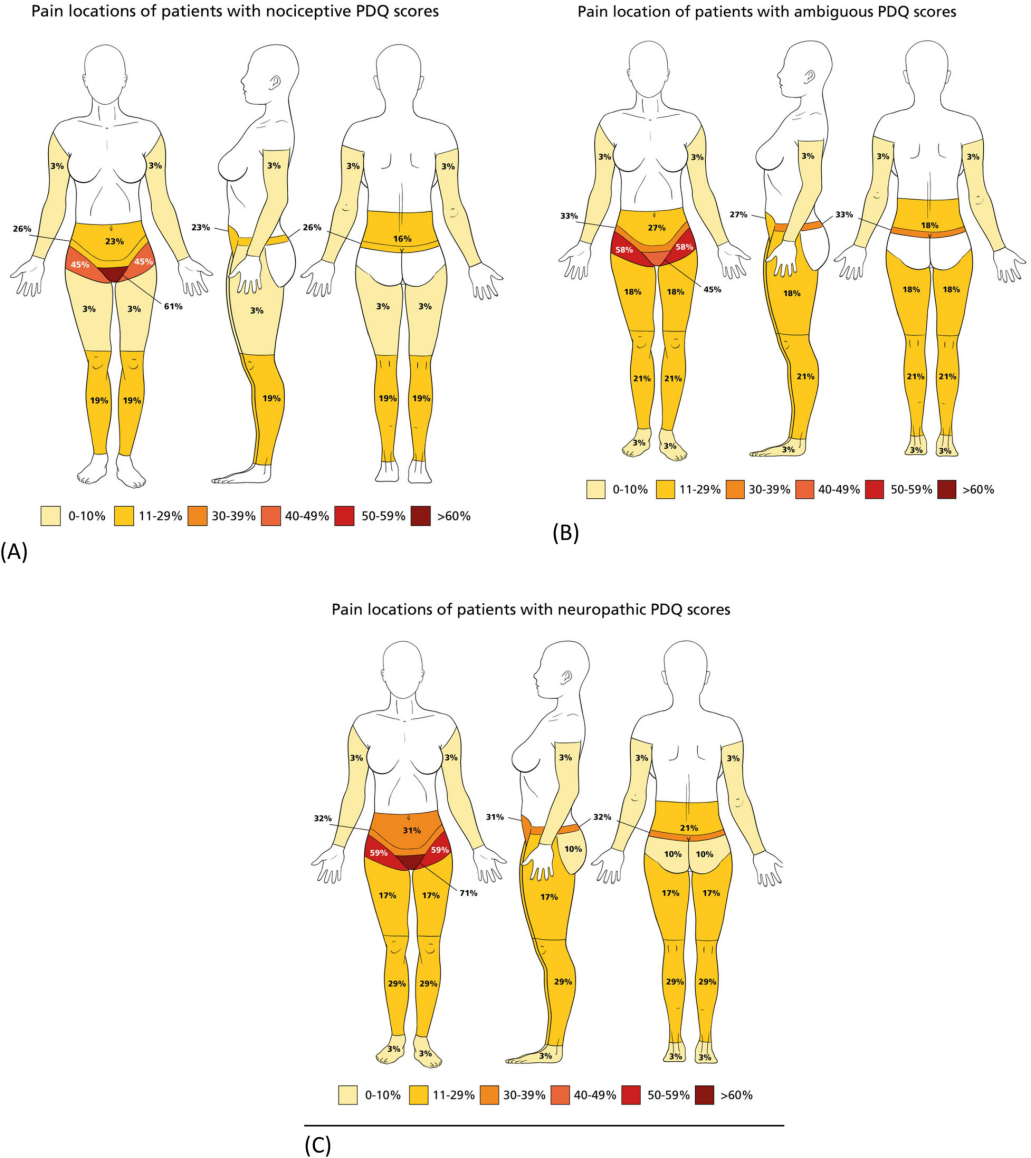
**(A)** Pain locations in participants with nociceptive PDQ scores. **(B)** Pain locations in participants with ambiguous PDQ scores. **(C)** Pain locations in participants with neuropathic PDQ scores.

#### Pain assessment with PDQ

Of the 280 patients with I-MAPS, 142 completed a PDQ. Over half of patients (55% *n* = 78/142) had a strongly neuropathic PDQ score. The remaining 22% (*n* = 31/142) and 23% (*n* = 33/142) had nociceptive and mixed nociceptive and neuropathic (ambiguous) pain mechanisms based on PDQ responses (Table 2; Figure 2).

**Table 2:** PDQ scores by type of mesh device.

Patient phenotype by PDQ	Total *n* = 142 (%)	Retropubic *n* = 69 (%)	TOT *n* = 73 (%)	*P* value
Nociceptive pain	31 (22%)	18 (26%)	13 (18%)	0.31^a^
Ambiguous pain	33 (23%)	17 (25%)	16 (22%)	0.84^a^
Neuropathic pain	78 (55%)	34 (49%)	44 (60%)	0.24^a^

a Fishers exact test.

Bold value is statistically significant.

Patients with trans obturator devices had a significantly higher mean PDQ score compared to those with retropubic devices.

##### Comparison of patients with nociceptive, ambiguous and neuropathic PDQ scores

###### Demographics

The demographics of patients with nociceptive, ambiguous and neuropathic PDQ scores were comparable (see Table 3). Patients within the neuropathic pain group had a higher relative deprivation score, with a deprivation score of 4. An IMD rank of 4, indicates deprivation is amongst the 10 highest within England.

**Table 3:** Demographic data according to PDQ score subgroup.

Patient demographics	Nociceptive PDQ (*n* = 31)	Ambiguous PDQ (*n* = 33)	Neuropathic PDQ (*n* = 78)	*P* Value
Age median (range) [IQR]	61 (46–61) [54–61]	61 (34–77) [51–62]	61 (44–83) [56–62]	0.66^a^
BMI median (range) [IQR]	30 (20–43) [25–36]	31 (19–42) [27–34]	31 (21–47) [27–34]	0.92^a^
IMD median (range) [IQR]	6 (1–10) [3–8]	6 (1–10) [3–8]	4 (1–10) [2–8]	0.22^a^
Smoking (%)	1 (3%)	3 (9%)	13 (17%)	0.13^b^
Pain conditions (%)	22 (71%)	17 (52%)	45 (58%)	0.27^b^
Autoimmune conditions (%)	3 (10%)	4 (12%)	3 (4%)	0.24^b^
Mood conditions (%)	3 (10%)	8 (24%)	14 (18%)	0.31^b^
Diabetes (%)	3 (10%)	3 (9%)	1 (1%)	**0** **.** **04** ^b^

a Fishers exact test.

b Mann–Whitney *U* test.

Bold value is statistically significant.

##### Body mapping of pain in patients with nociceptive, ambiguous and neuropathic PDQ scores

In patients with nociceptive pain scores, self-reported pain distributions were mostly localised to the pelvic region, with the highest proportion of pain reported in the vagina (61% *n* = 19/31), groin (45% *n* = 14/31) and hip region (26% *n* = 8/31). A smaller proportion of patients experienced radiation of pain in the thighs and lower leg (3% *n* = 1/31% and 19% *n* = 6/31) (See Figure 3A).

Neuropathic PDQ scores were associated with both localised pelvic pain and radiation to the buttocks (10% *n* = 8/78), thighs (17% *n* = 13/78) and lower legs (29% *n* = 23/78). There was no significant difference in pain radiation to the thighs (*p* = 0.12) and lower leg (*p* = 0.57) in patients with nociceptive, ambiguous and neuropathic pain scores. Buttock pain was significantly higher (*p* = 0.04) in patients with neuropathic pain (See Figure 3C).

#### Assessment of pain using e PAQ

Of the 280 patients, 203 completed an e PAQ assessment (see Table 4). Dyspareunia was associated with the highest reported pain scores however this was not significantly different across the 3 pain subgroups. There was no significant difference in e PAQ pain scores between patients with retropubic and trans obturator devices.

**Table 4:** Comparison of e PAQ assessments of pain, impact on QOL and health-related QOL measures (EQ5D) between patients with nociceptive, ambiguous and neuropathic PDQ scores.

Questionnaire domains	Total *n* = 203 (range)[IQR]	Nociceptive *n* = 31 (range)[IQR]	Ambiguous *n* = 33 (range)[IQR]	Neuropathic *n* = 78 (range)[IQR]	*P* value
e PAQ pain scores					
Bladder pain median (range) [IQR]	33 (0–100) [22–44]	33 (0–67) [11–44]	33 (11–67) [11–44]	43 (0–100) [22–56]	0.11^a^
Vaginal pain median (range) [IQR]	8 (0–100) [0–58]	8 (0–87) [0–50]	0 (0–100) [0–25]	21 (0–100) [0–60]	**0** **.** **03** ^a^
Dyspareunia median (range) [IQR]	75 (0–100) [33–92]	25 (0–100) [0–75]	92 (0–100) [25–100]	63 (0–100) [29–92]	0.21^a^
e PAQ QOL scores					
Bladder QOL median (range) [IQR]	73 (0–100) [33–100]	44 (0–100) [22–67]	78 (0–100) [33–100]	89 (0–100) [44–100]	**0** **.** **0005** ^a^
Bowel QOL median (range) [IQR]	33 (0–100) [11–67]	11 (0–100) [11–44]	28 (0–100) [0–53]	33 (0–100) [22–78]	0.06^a^
Vaginal QOL median (range) [IQR]	33 (0–100) [11–58]	33 (8–67) [13–46]	33 (8–83) [17–67]	42 (0–100) [21–60]	0.29^a^
Sexual QOL median (range) [IQR]	47 (0–100) [20–75]	17 (7–75) [9–61]	83 (0–100) [46–100]	77 (7–100) [56–92]	0.06^a^
EQ5D scores					
	Total score *n* = 65 (SD)	Nociceptive *n* = 16	Ambiguous *n* = 16	Neuropathic *n* = 33	*P* Value
Mobility (range) [IQR]	2 (1–5) [1–4]	3 (1–5) [1–4]	3 (1–5) [1–4]	3 (1–4) [1–4]	0.94^a^
Self-care (range) [IQR]	2 (1–4) [1–3]	1 (1–4) [1–2]	1 (1–4) [1–3]	2 (1–4) [1–3]	0.08^a^
Usual activities (range) [IQR]	3 (1–5) [2–4]	1 (1–4) [1–4]	3 (1–5) [2–4]	3 (1–5) [2–4]	**0** **.** **02** ^a^
Anxiety (range) [IQR]	3 (1–5) [2–4]	3 (1–5) [2–3]	3 (1–5) [1–3]	3 (1–5) [2–4]	0.56^a^
VAS health (range) [IQR]	53 (10–90) [40–70]	70 (35–90) [60–80]	50 (10–85) [40–70]	50 (10–90) [40–80]	0.13^a^
WHO 5 Index (range) [IQR]	20 (0–84) [12–84]	42 [16–56]	20 [12–34]	20 [12–34]	0.11^a^

a Kruskal–Wallis test.

Bold value is statistically significant.

#### Assessment of QOL

Bladder pain was the highest rated on QOL using the e PAQ questionnaire. Vagina and bowel pain were scored lowest on QOL impact.

Patients within the neuropathic pain sub-group generally reported higher pain scores and increased impact of pain on QOL. Vaginal pain was significantly higher in the neuropathic pain group (*n* = 0.03) and QOL related to vaginal pain symptoms were significantly impacted (*p* = 0.0005) in subjects with neuropathic pain compared to those with nociceptive pain (see Table 4).

Sixty-five patients completed the EQ5D and WHO 5 Index questionnaires. These findings are reported in Table 4. Patients within the neuropathic pain sub-group, generally reported reduced functional ability and reduced mental and physical well-being compared to those with nociceptive pain and a significant reduction in ability to undertake usual activities (Table 4). Subjects within all pain subgroups reported low mental well-being scores (<50) however the neuropathic and ambiguous pain subgroups reported scores consistent with depression (<28).

EQ5D: higher scores represent increased severity in each health dimension
A score of 1: no problems with these activities.
A score of 2: a slight problem.
A score of 3: a severe problem.
A score of 4: being unable to perform the activity at all.
WHO-5 and VAS Health scores: higher scores represent higher subjective mental and physical wellbeing.
WHO-5 scores of 50 or less indicates poor well-being, and a score of 28 or below suggests possible depression.

### Sub-group analysis: patients reporting pain beyond expected anatomical locations

Five patients reported pain beyond expected neuro-anatomical locations including the arms (*n* = 3) and the feet (*n* = 2).

Demographics of this patient subgroup were comparable to the study group (see Supplementary Table 1).

## Discussion

This cross-sectional study exploring the pain phenotypes of patients with I-MAPS identified that over half appeared to have neuropathic pain, based on PDQ assessment. This was supported by pain body mapping which revealed distribution of pain to be neuroanatomically plausible. Distant pain beyond the expected pain locations was reported in 2% (*n* = 5/280) of cases suggesting the possibility of an additional nociplastic pain component in some patients. These results indicate that I-MAPS is likely to involve mixed and overlapping pain subgroups of neuropathic, nociceptive and potentially nociplastic rather than a single pain component.

The I-MAPS study group exhibited high levels of non-pain symptoms including mental and functional impairment compared to U.K EQ5D population norms (16). These were particularly pronounced in patients within the neuropathic-pain subgroup compared to the nociceptive. This exemplifies the multidimensional impact and burden of chronic pain secondary to I-MAPS. Given the mixed pain phenotype and multisystem impact of I-MAPS, a multimodal approach directed at CNS rather than peripheral processes, are likely to be the most effective management approach. This is the first study in publication to examine the pain phenotypes involved in I-MAPS.

Knowledge of the underlying mechanisms behind this pain are lacking. Pain phenotyping provides several benefits. It supports understanding of the complexity and heterogeneity of pain through identification of pain related behaviours and symptoms. This is advantageous in that it allows patients to access, targeted and personalized treatment that comply with the global move towards “precision medicine” (17). Classification of pain through assignment of a label, is also recognised as providing psychological benefits through validation of patient's experience of pain and a diagnosis can empower patients to develop coping strategies (18).

Our study identified the dominant sub-group of patients with I-MAPS had a neuropathic phenotype. However, almost a quarter of patients had an ambiguous pain phenotype, and these may represent a mixed pain phenotype. Mixed pain types are considered more challenging to treat than predominant nociceptive or neuropathic pain sub-groups (19–21). Existing knowledge supports the heterogeneity of most chronic pain conditions, which are considered a complex interplay of nociceptive, neuropathic or mixed pathogenic mechanisms (22, 23). Some authors state that attempting to categorise pain as being confined to one mechanistic group is an oversimplification (24) and that most pain groups represent a mixed picture with substantial mechanistic overlap (25). Further research is required to evaluate if categorising pain phenotypes will enable clinicians to direct treatment options to improve outcomes for patients with I-MAPS.

A sub-set of patients had multi-site, distant and poorly localised pain related to their mesh device; 2% (*n* = 5/280) reported feet and upper limb involvement. This is considered to be highly suggestive of nociplastic pain (26). The phenotype nociplastic pain was introduced by the IASP in 2016 (27) to describe a third pain mechanism distinct from nociceptive and neuropathic pain characterised by a wide spatial distribution of pain with alterations in cognition, mood and heightened fatigue. Nociplastic pain can manifest in any chronic pain condition and can occur as a continuum with nociceptive and neuropathic pain.

Over half (52% *n* = 146/280) of patients within the I-MAPS study group had co-existing pain conditions including fibromyalgia, chronic back pain and Irritable Bowel Syndrome (IBS). There may be several explanations for this. Patients with I-MAPs may have pre-existing Chronic Overlapping Pain Conditions (COPC) and therefore experience widespread painful and multi-symptom disorders. These individual conditions may have been diagnosed in isolation without consideration for their systemic and interrelated nature. Another possibility is that chronic pain secondary to peripheral nerve injury by mesh implantation may have provoked a central pain syndrome through hyperexcitablility of the central nervous system resulting in Central Sensitization. Central Sensitization has been associated with several chronic pelvic pain conditions including endometriosis and Interstitial Cystitis and it is possible that I-MAPS may share a similar pain aetiology however further research is required to determine this.

Our I-MAPS study group demonstrated low mental wellbeing scores consistent with clinical depression. Depression has been theorized to amplify experience of pain through development of maladaptive thought processes resulting in increased activity in brain regions responsible for pain perception (28). Furthermore, chronic pain can result in sleep restriction and physical inactivity further modulating pain experience. Experience of trauma has also been attributed to development of chronic pain syndromes. Subjects with I-MAPS accessing our service describe experiences of trauma in relation to mesh with shared experiences of sleep disturbance, fatigue and pain (29). It is plausible that experience of I-MAPS is exacerbated by psychological trauma experienced in relation to mesh complications however further research within this area is required to establish the psychological impact of I-MAPS on patients.

This study has identified that I-MAPS appears to be a predominately neuropathic mediated pain process with overlapping components of nociceptive and possible nociplastic pain mechanisms.

Multi-centre, prospective studies are required in future studies to ensure the findings are representative of this patient cohort and to increase the external validity of the findings.

Further research is required to explore the psychological implications of I-MAPS through qualitative research. High quality and robust studies are required to explore the efficacy of non-surgical treatments over surgical management in the management of I-MAPS.

### Strengths

The strength of this study includes the large study size of 280 patients; this is likely to be one of the largest single centre cohorts of women with I-MAPS and therefore findings are more likely to be representative of this patient cohort. Other strengths include the use of validated questionnaires to assess pain and non-pain symptoms including the PDQ, EQ5D and e PAQ. This the first study in publication to explore the pain mechanisms involved in I-MAPS.

### Limitations

This study has several limitations. These include use of retrospectively collected data from a single centre and the absence of data on sensory pain thresholds. Questionnaires including the PDQ, e PAQ, EQ5D and WHO whilst validated questionnaires are not validated for use in patients experiencing mesh complications. There are currently no validated questionnaires for this patient population; the APPRAISE study is expected to develop research tools relevant to mesh affected women (30).

Our study presents EQ5D data from only 65 participants. The EQ5D questionnaire was only introduced as a mandatory questionnaire by NHS England in 2023; patients seen before this period hence had missing data relevant to this and was hence an unavoidable limitation. The small study sub-group of 5 with distant pain meant there was limited generalisable conlusions that could be drawn from these findings. These results are intended to encourage further work in this area.

## Conclusion

Our study identified I-MAPS to be of predominately neuropathic origin with mixed nociceptive and possible nociplastic components.

## Data Availability

The raw data supporting the conclusions of this article will be made available by the authors, without undue reservation.
