# Supplementary material for: Phenotyping of isolated mesh associated pain secondary to continence mesh device insertion
Source: Front Pain Res (Lausanne). 2026 Jun 17;7:1793212. doi: 10.3389/fpain.2026.1793212 (PMC13318887; doi:10.3389/fpain.2026.1793212)
Supplement: Supplementary file 1 [file Table1.docx]

Supplementary

Table 1: A comparison of demographic, pain assessment, e PAQ and EQ5D scores for patient sub-group reporting distant pain against the study group

|  | Total (n=275) | Patients reporting distant pain (n=5) |
| --- | --- | --- |
| Age median (range) [IQR] | 60 (33-94) [57-60] | 61 (61-64) [61-63] |
| BMI median (range) [IQR] | 30 (19-47) [26-34] | 29 (23-38) [25-38] |
| IMD median (range) [IQR] | 5 (1-10) [3-8] | 4 (3-8) [3-8] |
| Smoking (%) | 26 (9%) | 0 |
| Pain conditions (%) | 143 (52%) | 3 (60%) |
| Autoimmune conditions (%) | 25 (9%) | 0 |
| Mood conditions (%) | 45 (16%) | 0 |
| Diabetes (%) | 22 (8%) | 1 (20%) |
| Pain assessments |  |  |
| PDQ score n=142 (range) [IQR] | 20 (0-38) [14-25] | 21 (8-28) [10-38] |
| VAS pain score (range) [IQR] | 7 (0-10) [5-8] | 9 (6-9) [10-27] |
| e PAQ pain scores |  |  |
| Bladder pain (range) [IQR] | 33 (0-100) [22-44] | 28 (11-44) [11-44] |
| Vaginal pain (range) [IQR] | 8 (0-100) [0-58] | 25 (0-42) [0-42] |
| Dyspareunia (range) [IQR] | 75 (0-100) [33-92] | 92 (83-100) [83-100] |
| EQ5D |  |  |
| Mobility (range) [IQR] | 2 (1-5) [1-4] | 3 (3-5) [3-5] |
| Self-care (range) [IQR] | 2 (1-4) [1-3] | 2 (1-2) [1-2] |
| Usual activities (range) [IQR] | 3 (1-5) [2-4] | 4 (4-4) [4-4] |
| Anxiety (range) [IQR] | 3 (1-5) [2-4] | 3 (3-4) [3-4] |
| VAS health (range) [IQR] | 53 (10-90) [40-70] | 25 (10-25) [10-25] |
| WHO 5 Index (range) [IQR] | 20 (0-84) [12-84] | 12 (4-28) [10-25] |
